# Comparative Analysis of Single Lateral Locked Plate and Double Locked Plate Application in the Treatment of Bicondylar Tibial Plateau Fractures

**DOI:** 10.7759/cureus.19298

**Published:** 2021-11-05

**Authors:** Ceyhun Çağlar, Serhat Akcaalan, Halil İbrahim Özaslan, Merve Bozer, Fahri Emre, Mahmut Uğurlu

**Affiliations:** 1 Department of Orthopaedics and Traumatology, Ankara City Hospital, Ankara, TUR; 2 Department of Orthopaedics and Traumatology, Ankara Yildirim Beyazit University, Ankara, TUR; 3 Department of Orthopaedics and Traumatology, Ankara Gulhane Training and Research Hospital, Ankara, TUR

**Keywords:** posterior tibial slope, medial proximal tibial angle, condylar width, orif, tibial plateau fractures

## Abstract

Background and objective

Bicondylar tibial plateau fractures (BTPFs) have been controversial in terms of surgery planning, due to articular joint surface comminution, severe soft tissue injury, and the risk of complications. The aim of this study was to conduct a clinical, functional, and radiologic comparison of the dual locked plate (DLP) and single lateral locked plate (SLLP) techniques.

Methods

Retrospectively analysed were 54 patients who underwent surgical treatment with DLP or SLLP due to the diagnosis of BTPFs, between January 2018 and June 2020. Patients were evaluated in the clinic with regard to their demographic characteristics, mechanisms of injury, follow-up periods, measurement of the range of motion degrees, functional scores, and radiographic parameters. The Knee Injury and Osteoarthritis Outcome Score (KOOS), Lysholm Knee Score (Lysholm) and Oxford Knee Score (OKS) were chosen as the functional scores. The condylar width, medial proximal tibial angle (MPTA), posterior tibial slope (PTS) and fracture union time were calculated radiographically.

Results

The patients in the DLP group achieved significantly higher scores for all three scales when the KOOS, Lysholm, and OKS, respectively (P = 0.008, P = 0.048, P = 0.006), were compared. Radiographically, the mean increase in the condylar width of 1.72 mm in the DLP group and 2.59 mm in the SLLP group was measured (P = 0.010, P = 0.010, respectively). The mean decrease in MPTA was 1.75° in the DLP group and 3.54° in the SLLP group, which was statistically significant (P = 0.005, P = 0.001, respectively). An increase in the posterior tibial slope was measured at a mean of 1.8° in the DLP group and 1.4° in the SLLP group (P = 0.001, P = 0.008, respectively). On the other hand, when the condylar width, MPTA and PTS between the DLP and SLLP groups were compared, no significant difference was found (P = 0.179, P = 0.247, P = 0.611, respectively).

Conclusion

Better results were obtained in patients who underwent the DLP procedure when compared to those who had the SLLP. There was no radiographic difference between the two surgical procedures. Although DLP is an effective and reliable method in the treatment of BTPFs, the SLLP procedure also provides satisfactory results in patients with appropriate indications.

## Introduction

Tibial plateau fractures can be categorized according to the Schatzker or AO-Müller/Orthopaedic Trauma Association (AO/OTA) classifications, and they constitute approximately 30% of all tibial fractures [1,2]. Bicondylar tibial plateau fractures (BTPFs), which are type V/VI and type 41-C, respectively, according to the Schatzker and AO/OTA classifications, constitute approximately 18% to 39% of all tibial plateau fractures, and are complex and serious injuries [3,4].

In recent years, with the increase in traffic accidents, which cause high-energy injuries, the number of BTPFs has increased in correlation [5]. BTPF treatment is a big challenge for orthopedic surgeons due to problems such as excessive comminution on the articular surface, and severe soft tissue injury and its vulnerability to complications [6,7]. The main purpose of surgery is the anatomical restoration of the joint surface, avoidance of soft tissue damage, and early mobilization of the patient by ensuring the alignment of the lower extremities [8].

Many different methods, such as external fixators, Ilizarov circular frame, and open reduction internal fixation (ORIF) are performed in the surgical treatment of BTPFs. The open reduction and dual locked plate (DLP) method is one of the ideal surgical procedures, with proven biomechanical stability, since it supports both lateral and medial fragments stably [9,10]. However, the biggest handicap of this method is that wide soft tissue dissection at the fracture site increases the possibility of wound complications, especially in patients with severe soft tissue injury [9]. With the development of modern locking plating systems and the use of the minimally invasive percutaneous osteosynthesis (MIPO) technique, a good alternative to BTPF treatment with a single lateral locked plate (SLLP) has emerged in patients with severe soft tissue injury. In this technique, the possibility of secondary reduction loss, especially in the medial fragments, appears to be a disadvantage [11,12].

The aim of this study was to compare the clinical, functional, and radiological outcomes of the DLP and SLLP procedures, which are the two preferred methods in the treatment of BTPFs.

## Materials and methods

Retrospectively analysed were 54 patients who underwent surgical treatment with the DLP or SLLP procedure due to the diagnosis of BTPFs between January 2018 and June 2020, in our clinic, and met the inclusion criteria, with the inclusion criteria being between 18 and 80 years of age, and having had a diagnosis of Schatzker type V/VI BTPF for which they were operated on using either the DLP or SLLP procedure. Open fractures, pathological fractures, additional fractures of the same extremity, neurovascular injuries, soft tissue ligament and meniscus injuries with fracture, having been operated on for other surgical procedures, and not having attended regular clinical follow-ups were accepted as exclusion criteria.

This study was approved by Ethics Committee Nr. 2 of Ankara City Hospital (approval number E2-21-334). Informed consent was obtained from all patients included in the study.

Surgical procedure

All of the patients were operated on in the supine position and a pneumatic tourniquet was used to control bleeding. All patients were operated by the same orthopedic surgeon. The SLLP or DLP procedure was not randomized. The SLLP procedure was preferred in cases of large and non-displaced medial fragment, medial condyle in bone-contact, lack of fractures in the coronal plane and lack of osteoporosis [5,8]. In patients with severe soft tissue injury, the affected extremity was splinted. Soft tissue edema was expected to decrease by elevation and ice application. The mean time from injury to surgery in patients was 5.2 ± 3.7 days. Surgery was performed after soft tissue healing.

Single Lateral Locked Plate

**Figure 1:**
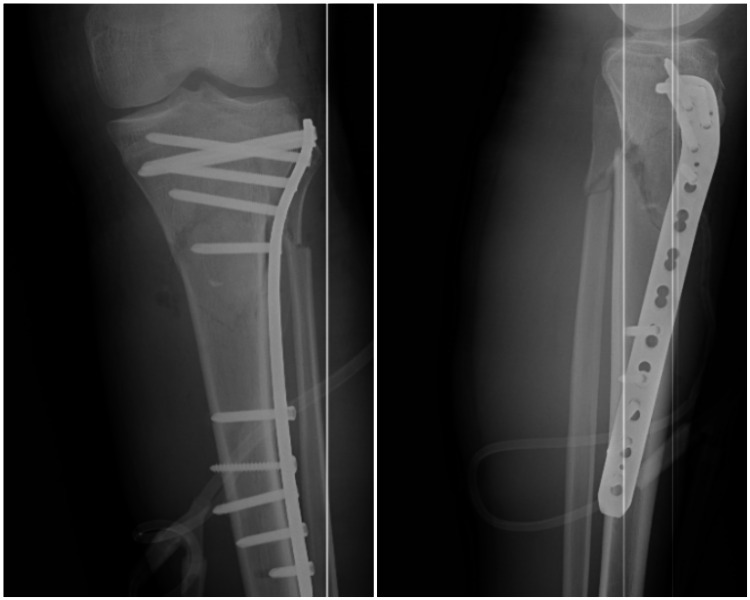
Anteroposterior and lateral radiographs of a patient who was treated using the single lateral locked plate due to Schatzker type VI bicondylar tibial plateau fracture

The standard anterolateral approach was used in the proximal tibia in patients who were operated on using this procedure. The lateral meniscus was lifted with a suture after horizontal capsulotomy to provide joint vision. After the anatomic restoration of the joint was achieved, it was temporarily fixed with K-wires. In the case of bone defect, the defect was filled with an allograft. Next, a SLLP of appropriate length for the fracture pattern was placed using the MIPO technique. A separate mini incision was made to the distal of the plate so that the screws could be applied to the distal of the fracture. Final fracture fixation was completed by placing screws of appropriate length. After fluoroscopic control, the capsule was repaired and the operation was terminated (Figure 1).

Dual Locked Plate

**Figure 2:**
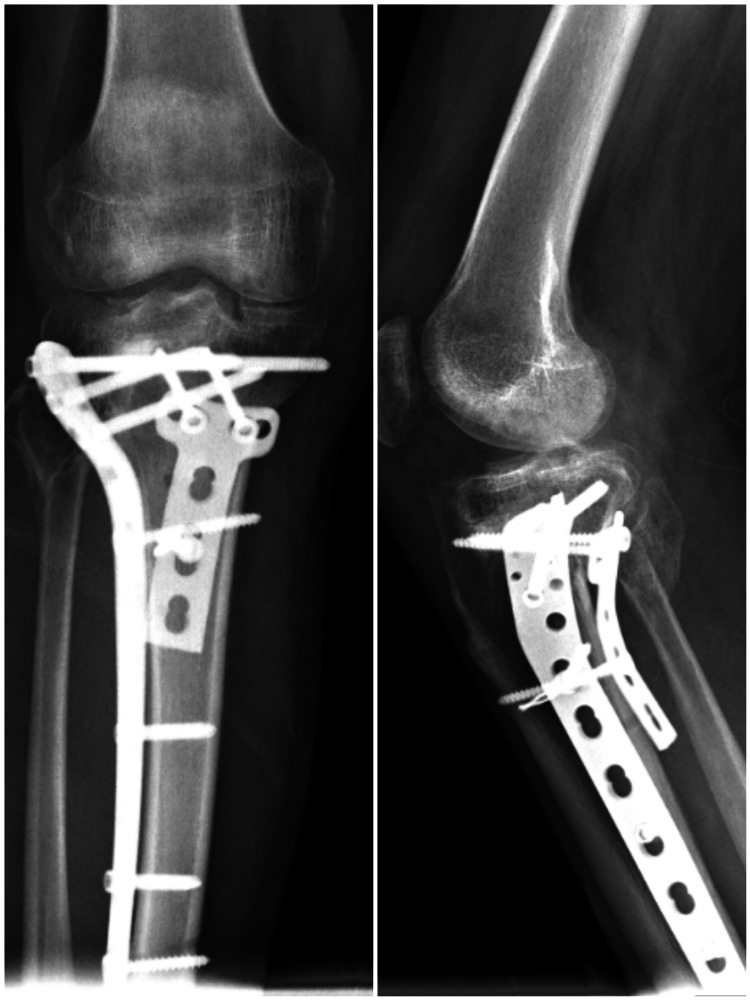
Anteroposterior and lateral radiographs of a patient who was treated using the dual locked plate due to Schatzker type VI bicondylar tibial plateau fracture

In this technique, in addition to patients with a SLLP, a posteromedial approach was made from the proximal tibia at least 5 cm distal to the anterolateral incision. After the SLLP procedure was completed, the DLP procedure was started. During the posteromedial approach, the patient's leg, which is in the supine position, is placed in a figure four position to facilitate the approach. The interval between the medial head of the gastrocnemius muscle and the semimembranosus muscle was explored. When necessary, the pes anserinus was separated from the adhesion site and posteromedial tibia was seen. Joint reduction was achieved by elevating the collapsed posteromedial fragment. In the case of bone defect, the defect was filled with an allograft. Next, additional fixation was made using another locking plate suitable for the anatomy and fracture pattern of this region. After fluoroscopic control, the pes anserinus was sutured instead of the adhesion and the operation was terminated (Figure 2).

Rehabilitation

All patients were given an angle adjustable knee brace postoperatively, regardless of which surgical procedure was performed. All patients were observed in the hospital until their soft tissue injuries healed. Extremity edema was prevented by frequent elevation and ice application, especially in the first 24 hours after surgery. All patients were regularly trained by an experienced physiotherapist. While 0°-30° flexion range of motion (ROM) was allowed in the early postoperative period, the degree of flexion was gradually increased to 120° in the sixth week. All patients were given partial weight-bearing at the 8th week and full weight-bearing at the 12th week postoperatively.

Clinical and functional assessment

All of the patients were evaluated by the same doctor at the clinic one year post-surgery. The patients were evaluated functionally using Knee Injury and Osteoarthritis Outcome Score (KOOS), Lysholm Knee Score (Lysholm) and Oxford Knee Score (OKS). Moreover, the degree of knee extension-flexion and ROM were measured by the same observer using a goniometer and the ROM of the patients was measured and recorded.

Radiographical assessment

**Figure 3:**
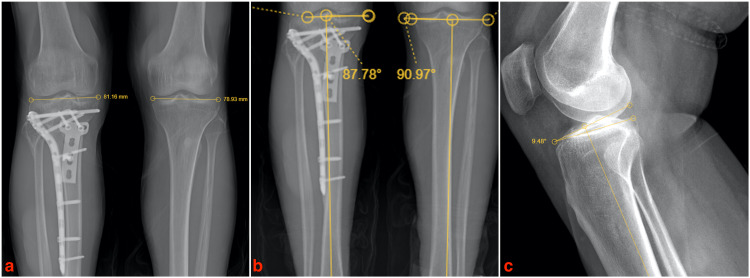
Measurement of the radiographic parameters used to evaluate the patients. (a) Measurement of the condylar width in both knees and the difference between them, (b) measurement of the medial proximal tibial angle in orthoroentgenogram and the difference between them and (c) measurement of the posterior tibial slope angle

Orthoroentgenogram, anteroposterior (AP) and lateral radiographs of both knees were obtained at one year post-surgery. Both the operated and contralateral condylar widths of the patients were measured in the knee AP radiographs and the difference between them was calculated (Figure 3, panel a). A difference in the condylar width between 0 and 5 mm was considered satisfactory [13]. Both the operated and contralateral medial proximal tibial angle (MPTA) of the patients were measured in the orthoroentgenogram and the difference between them was calculated (Figure 3, panel b). The MPTA range of 87° ± 5° in the coronal plane was considered an acceptable result [14]. In addition, both the operated and contralateral posterior tibial slope (PTS) of the patients were measured in the knee lateral radiographs and the difference between them was calculated (Figure 3, panel c). The 9° ± 5° posterior slope of the proximal tibia in sagittal alignment was evaluated as successful [15]. In addition, the radiographic union times of all of the patients were calculated. Radiographically, the union of three out of four cortices was considered as a radiographic union.

Statistical analysis

All of the statistical analyses were performed using IBM SPSS Statistics 24.0 for Windows 15.0 (IBM Corp., Armonk, NY). The conformity of the data to normal distribution was evaluated with the Kolmogorov-Smirnov test and it was seen that all data were compatible with normal distribution. The independent sample t-test, a parametric test, and the Mann-Whitney U test, a non-parametric test, were applied and the data obtained for both groups were compared statistically. P < 0.05 was considered statistically significant.

## Results

The demographic characteristics, mechanisms of injury, and follow-up periods of the patients are shown in Table 1. It was observed that the patients operated on for BTPFs were predominantly male and young. When the mechanisms of injury of these patient groups were evaluated, it was noted that the patients were generally injured after high-energy traumas. Both of the surgical technique groups had a follow-up period of more than one year.

**Table 1:** Demographic characteristics of patients n: number of patients; DLP: dual locked plate; SLLP: single lateral locked plate

		DLP	SLLP
Gender (n)	Male	20	16
	Female	8	10
Mean age (years)		39.30 ± 8.96	44.57 ± 10.46
Injury mechanism (n)	In-vehicle traffic accident	6	6
	Non-vehicle traffic accident	8	4
	Bicycle accident	2	5
	Fall from a height	8	5
	Simple fall	2	5
	Crush injury	2	1
Follow-up time (months)		16.14 ± 7.86	14.31 ± 6.45

The data obtained from the patients, with their clinical and functional scores, are presented in Table 2. Patients who underwent both surgical procedures were evaluated using the KOOS, Lysholm, and OKS, respectively, and scores were found in favor of the DLP for all three scales (P = 0.008, P = 0.048, P = 0.006). When the operated knee ROM values of the patients were compared, it was found that significantly higher ROM was obtained in the DLP group compared to the SLLP group (P = 0.045).

**Table 2:** Comparison of both treatment groups in terms of clinical and functional parameters KOOS: Knee Injury and Osteoarthritis Outcome Score; OKS: Oxford Knee Score; LKS: Lysholm Knee Score; ROM: range of motion; DLP: dual locked plate; SLLP: single lateral locked plate; SD: standard deviation Data are presented as means ± SDs (min-max). *Statistically significant.

	DLP	SLLP	P
KOOS	88.00 ± 6.63 (72-99)	82.64 ± 7.49 (66-95)	0.008*
OKS	43.23 ± 3.36 (37-48)	41.10 ± 4.38 (30-48)	0.048*
LKS	91.53 ± 6.54 (73-99)	85.14 ± 8.41 (68-98)	0.006*
Flexion (°)	124.23 ± 6.73 (100-130)	120.53 ± 8.31 (100-130)	0.017*
Extension (°)	2.88 ± 3.78 (0-15)	5.00 ± 5.09 (0-20)	0.136
Knee ROM (°)	121.34 ± 7.55 (100-130)	115.53 ± 12.64 (80-130)	0.045*

Radiological measurements of the operated and contralateral tibial plateaus of the patients who were operated on using both surgical procedures are shown in Table 3. The mean increase in the condylar width was 1.72 mm in the DLP group and 2.59 mm in the SLLP group and a significant difference was found (P = 0.010, P = 0.010, respectively). The mean decrease in MPTA was 1.75° in the DLP group and 3.54° in the SLLP group, which was statistically significant (P = 0.005, P = 0.001, respectively). An increase in the PTS was measured at a mean of 1.8° in the DLP group and 1.4° in the SLLP group, and again, a significant difference was observed (P = 0.001, P = 0.008, respectively).

**Table 3:** Radiographical measurements of operated and contralateral tibial plateaus of patients who underwent one of two different surgical procedures DLP: dual locked plate; SLLP: single lateral locked plate; SD: standard deviation Data are presented as means ± SDs (min-max). *Statistically significant.

		Contralateral tibial plateau	Operated tibial plateau	P
DLP	Condylar width (mm)	81.85 ± 5.25 (69.87-89.21)	83.57 ± 5.51 (70.09-92.65)	0.010*
DLP	Medial proximal tibial angle (°)	88.23 ± 4.18 (84.85-90.38)	86.48 ± 6.31 (81.43-88.62)	0.005*
DLP	Posterior tibial slope (°)	10.49 ± 2.75 (5.22-15.28)	12.29 ± 3.50 (4.41-18.27)	0.001*
SLLP	Condylar width (mm)	81.66 ± 3.35 (77.93-90.62)	84.25 ± 4.27 (77.65-94.36)	0.010*
SLLP	Medial proximal tibial angle (°)	88.45 ± 4.76 (84.19-91.27)	84.91 ± 8.33 (76.34-90.48)	0.001*
SLLP	Posterior tibial slope (°)	12.59 ± 2.24 (8.77-16.34)	13.99 ± 2.95 (5.28-18.75)	0.008*

The measurement differences between the operated and contralateral tibial plateaus of the patients undergoing both surgical procedures are presented in Table 4. The differences in the condylar width, MPTA and PTS, respectively, were compared between both surgical techniques and no statistically significant difference was found (P = 0.179, P = 0.247, P = 0.611).

**Table 4:** Comparison of radiographic measurement differences in two different surgical techniques DLP: dual locked plate; SLLP: single lateral locked plate; SD: standard deviation Data are presented as means ± SDs (min-max).

	DLP	SLLP	P
Condylar width difference (mm)	1.76 ± 2.29 (-1.61-5.93)	2.59 ± 2.16 (-1.85-5.90)	0.179
Medial proximal tibial angle difference (°)	1.75 ± 2.85 (-2.36-6.02)	3.54 ± 4.56 (-4.48-8.17)	0.247
Posterior tibial slope difference (°)	1.75 ± 2.44 (-3.07-5.97)	1.40 ± 2.59 (-4.08-4.85)	0.611

The two groups were compared by looking at another radiographic parameter, the duration of fracture union. While the mean radiographic union time was 12.64 ± 3.49 weeks in the DLP group, it was 13.74 ± 4.15 weeks in the SLLP group. There was no statistically significant difference between the two surgical techniques (P = 0.455).

Radiographic union was achieved in all of the patients. During the follow-up, reduction loss was observed in three patients in the SLLP group and one patient in the DLP group, but no additional surgery was performed as it was not clinically symptomatic. Superficial infection developed in the immediate postoperative period in two patients in the DLP group. Immediate wound debridement and intravenous (i.v.) antibiotherapy were applied to the patients. The infections were treated without the need for implant removal. In the SLLP group, deep tissue infection occurred in one patient in the late period. Since the patient had fracture healing, implant removal and wound debridement were performed and i.v. antibiotherapy was administered. After one month of treatment, the soft tissue infection had been treated before osteomyelitis had developed.

## Discussion

Although Schatzker type V/VI BTPFs are always a big challenge for orthopedic surgeons, many different methods are still being discussed regarding the ideal treatment method. According to Georgiadis, dual plate fixation is the gold standard method in Schatzker type V/VI BTKFs [16]. Yoo et al. found that the SLLP technique was insufficient in fixing the posteromedial fragment. Therefore, they argued that the medial plate should be added to the BTPF treatment [17]. To support these, Higgins et al. and Egol et al., in their biomechanical analyses, showed that patients who were treated using DLP after cycling loading tests had less collapse in the medial plateau than patients who were treated using SLLP [12,18]. According to Yao et al. and Weaver et al., SLLP treatment is sufficient and safe if the BTPFs meet the criteria of tibial condyles in bone contact, simple fractures in the sagittal plane, and large, non-marginal medial fragments [5,19]. In this study, the DLP and SLLP treatments, which are among the frequently preferred internal fixation methods in the clinic, were compared by considering many parameters.

Different scoring systems have been defined and preferred in order to evaluate the functional results of tibial plateau fractures. Citak et al. evaluated 20 patients operated on for Schatzker type V/VI BTPFs using the Knee Society Score (KSS) and Rasmussen functional score, and they did not see a significant difference between the patient results [20]. Menghi et al. followed up 38 patients operated on for BTPFs for a minimum of one year, and evaluated the patients who were treated using single and double plates, in terms of functionality, with the KOOS and 36-Item Short Form Health Survey, and reported that satisfactory results were achieved in both groups. They could not find a significant difference between the groups [21]. Pun et al. followed up 21 patients who were treated using single or double plates for a type C tibial plateau fracture for a minimum of 12 months, and used the Western Ontario and McMaster Universities Osteoarthritis Index questionnaire to evaluate the patients, and they determined that there was no significant difference between the two groups [22]. In the current study, the patients who were treated were evaluated using the KOOS, Lysholm, and OKS, and three different scales were preferred. In all three scales, unlike the other studies, significantly better scores were obtained in the group treated with the DLP. Although there was a significant difference between them, in both groups, excellent results were obtained according to the OKS and KOOS, and good results were obtained according to the Lysholm. Additional stability provided better functional scores in the patients who were treated using the DLP.

ROM is one of the most frequently used parameters to evaluate the functional status of patients after BTPFs. In the study conducted by Lee et al., 38 patients who were operated on for BTPFs were divided into three groups, as patients who received a single-plate, a classical double-plate, or hybrid double-plate treatment, and no significant difference was found between the three groups in terms of ROM [9]. In another study, 54 patients who were treated using a double buttress plate due to BTPFs were followed up for a minimum of one year, and an average of 107.6° flexion was achieved in the knee joint [23]. In another study, 20 patients who were treated using single and double plates were evaluated in terms of ROM, and 120° flexion was reached in the single-plate group, while 119° in the double-plate group, with no significant difference between them [20]. Zhao et al. reported a mean extension of 3.4° and flexion of 130° in 11 patients who were treated using double plate due to BTPFs [24]. In the current study, although functionally satisfactory ROM values were determined with both surgical procedures, significantly higher flexion and ROM values were obtained in the DLP group. As with the functional scores, bilateral stability directly affected postoperative rehabilitation.

Radiographically, anatomic joint reduction and proper alignment of the lower extremities are critical for early mobilization and creation of a functional knee joint [25]. In the current study, the condylar width and PTS of the operated knees were compared with those of the contralateral knees in both the DLP and SLLP groups. In both treatment groups, the condylar width and PTS were significantly increased and MPTA was significantly decreased when compared to the contralateral knee. Although no complete radiographic anatomic reconstruction could be achieved in either surgical procedure, the values obtained were very close to those of the contralateral knee and were within acceptable limits.

On the other hand, there are studies in the literature comparing DLP- and SLLP-treated BTPFs with radiographic parameters. Arouca et al. compared the coronal alignment of the medial proximal tibia, sagittal alignment of proximal tibia, and condylar width in radiographs taken in the immediate postoperative and late postoperative periods in 63 patients who were treated with DLP or SLLP, and did not observe a significant difference between the two treatment groups [26]. In another study, both surgical methods were compared by measuring the tibiofemoral anatomic angle, MPTA, and proximal posterior tibial angle parameters, and no significant difference was found [20]. Chang et al. reported in their meta-analysis that there was no difference between the malalignment, malreduction, and secondary loss of reduction in either treatment group [27]. However, Neogi et al. observed a higher varus collapse rate in cases where a single plate was applied [28]. In the current study, similar to the literature, no significant difference was found between the two surgical procedures in terms of the condylar width, MPTA and PTS differences that occurred when compared to the contralateral knees. Both treatment groups exhibited good results in radiographic reconstruction. Similarly, radiographic reduction loss occurred in four patients (three patients SLLP, one patient DLP) in both groups, but it was not functionally significant.

Although rare, nonunion can be seen after BTPFs. In one study, approximately, a 4% nonunion rate was reported in follow-up after BTPFs [18]. Causes such as injury to the nutrient vessels, bone defects, and the lack of stable fixation have been reported as the cause of nonunion [25]. In the current study, union was achieved in all of the patients and nonunion was not observed. The stable fixation that was applied and filling the bone defects with an allograft minimized the nonunion rate.

Soft tissue injury after BTPFs is an issue that should be considered for orthopedic surgeons. In some cases, treatment modalities may change according to the soft tissue injury. Baeri et al. reported that 7 of 83 patients who underwent ORIF for BTPFs developed deep infections, and they required 3.3 additional operations on average [29]. According to a systematic review and meta-analysis, it was observed that external fixation was not superior to internal fixation in the treatment of BTPFs in terms of soft tissue complications [30]. Chang et al. reported that single plate was not superior to dual plate in terms of soft tissue complications [27]. In the study conducted herein, superficial infection was detected in two patients in the DLP group, and deep tissue infection was detected in one patient in the SLLP group, and all were treated without complications. Whichever technique is used in the surgical treatment of BTPFs, soft tissue should be carefully evaluated before surgery and minimal dissection should be performed during surgery in order to minimize soft tissue injury.

This study has some limitations. First, it was a retrospective study and there were no preoperative data on the patients. Second, the questionnaires filled out for functional evaluation were based on patient statements and may not have given sufficient objective data. Third, although the radiological measurements and joint ROM measurements were made by the same doctor, there may have been measurement errors depending on the person. Fourth, in the radiographic measurements, both knees of the patients were accepted as the same size and calculations were made accordingly, whereas there may have been differences in size between each knee. Finally, although the number of patients and follow-up time were relatively sufficient, clearer results can be obtained with a larger number of patients and longer follow-up time.

## Conclusions

When the two different ORIF techniques used most frequently in BTPF treatment were compared, better functional results were obtained in patients who were treated using DLP compared to SLLP. Although postoperative rehabilitation was performed in the same way in both patient groups, better ROM values ​​were observed in the DLP group. No matter how successfully both treatment procedures are applied, there is a difference in condylar width, MPTA and PTS values compared to the contralateral knee. Radiographically, although an increase occurred in the condylar width and PTS and a decrease occurred in the MPTA with both treatments, there was no radiographic difference between the two surgical procedures. Although DLP is an effective method in the treatment of BTPF and a more reliable method in more complex fractures, the SLLP procedure also provides satisfactory results in patients with appropriate indications.
